# Association between the First Occurrence of Allergic Rhinitis in Preschool Children and Air Pollution in Taiwan

**DOI:** 10.3390/ijerph13030268

**Published:** 2016-02-27

**Authors:** Hui-Ying Chung, Chia-Jung Hsieh, Chun-Chieh Tseng, Lih-Ming Yiin

**Affiliations:** 1 Department of Public Health, Tzu Chi University, 701, Sec. 3, Zhongyang Rd., Hualien City 97062, Taiwan; fatty99@gms.tcu.edu.tw (H.-Y.C.); cjhsieh@mail.tcu.edu.tw (C.-J.H.); tsengcc@mail.tcu.edu.tw (C.-C.T.); 2 TCU Center for Value-Added Health Data Analysis and Application, 701, Sec. 3, Zhongyang Rd., Hualien City 97062, Taiwan

**Keywords:** air pollution, allergic rhinitis, nitrogen oxides, preschool children, traffic emission

## Abstract

The occurrence of allergic rhinitis (AR) may be significantly influenced by air pollution. This study examined the association between the first occurrence of AR in preschool children and the pre-incident levels of air pollutants in Taiwan. We identified 9960 eligible subjects from a systematic sampling cohort database containing 400,000 insureds of the National Health Insurance from 2007 to 2011 and matched them with the environmental monitoring data from 2006 to 2011 according to the locations of their clinics. Pre-incident levels were determined using the average concentrations of air pollutants one or two weeks prior to the AR diagnoses. Logistic regression analyses were performed to determine any significant relationships between AR and specific air pollutants. The first AR incidence for Taiwanese preschool children, which increased with age, was 10.9% on average; boys appeared to have a higher percentage (14.2%) than girls (8.27%). Among the air pollutants, carbon monoxide (CO) and nitrogen oxides (NO_X_) were significantly related to AR after adjusting for age and gender (*p* < 0.05). Because both pollutants are considered to be traffic emissions, this study suggests that traffic emissions in Taiwan need to be controlled to lower the prevalence of children’s AR.

## 1. Introduction

Allergic rhinitis (AR) is a common respiratory disease occurring in individuals who breathe air with allergens that cause nose inflammation and swelling. A variety of allergens can trigger AR, including molds, pollens, animal dander, and dust mites [1]. Seasonal changes, such as temperature and humidity variations, may also play a role in exacerbating AR [2,3,4]. When the nose suffers from AR, it becomes blocked, which likely also blocks the sinuses (sinusitis); both are irritating and cause discomfort and headaches, all of which may interfere with daily life. Although AR is usually not considered a severe disease, its potential to develop into asthma is of great concern and warrants investigation [5].

A number of studies have indicated that the increasing prevalence of AR is associated with air pollution severity [2,6,7,8,9,10,11,12]. Of the commonly monitored air pollutants, which include carbon monoxide (CO), nitrogen oxides (NO_X_), ozone (O_3_), particulate matter (PM), and sulfur dioxide (SO_2_), several have been found to be related to AR; however, the relationships are not consistent. For example, NO_2_, PM_10_ (aerodynamic diameter ≤ 10 μm) and SO_2_ have been associated with AR in China [11,13,14], whereas in the United States, O_3_ and PM_2.5_ (aerodynamic diameter ≤ 2.5 μm) are the related environmental factors [7]. Despite the differences in species, traffic-related air pollution or residence in an urban area appears to be an important factor for the increasing prevalence of AR in many countries [4,7,15,16,17,18,19,20].

Many studies have focused on the relationships between AR and the environment; however, not all age groups are studied evenly. There are relatively few data for preschool children, likely due to their low prevalence of AR or their limited ability to independently fill out research questionnaires. Nevertheless, preschool children’s health warrants additional attention and care, and this issue is relevant for discussion and investigations. Thus, we conducted this study in preschool children using Taiwan’s National Health Insurance Data, from which the AR subjects were identified by doctor’s diagnoses, and combining the environmental data from the subjects’ adjacent monitoring sites to determine the association between the first occurrence of AR and environmental pollutants.

## 2. Materials and Methods 

### 2.1. Inclusion of Subjects

We used the systematic sampling cohort database of 400,000 insureds of the National Health Insurance of Taiwan from 2007 to 2011 and found 23,103 preschool children aged 0–6 years. After excluding those who did not have sufficient information or adjacent environmental monitoring data, there were 9960 children, of which 4456 were male and 5504 were female. The diagnoses were made following the International Classification of Diseases, 9th Revision, Clinical Modification (ICD-9-CM), and the codes 477.0, 477.1, 477.2, 477.8 and 477.9 were categorized as AR. Those who were assigned to any of the ICD-9CM codes were selected as study subjects, and only the first diagnoses were used to relate to the air pollution at one and two weeks prior to them. Each eligible subject must have had more than one AR-related visit and been confirmed to be AR-free for at least one year prior to the first AR diagnosis during the period; that is, the year of 2007 was considered a clearance period from which no AR records were retrieved. There were 7326 AR-related visits, of which 1088 children, 632 male and 456 female, were eligible and included in the study. Because of the secondary data with the removal of personal identification, the study was granted for exempt review by the Research Ethics Committee of Tzu Chi General Hospital (No: IRB102-57, approved on 23 April 2013).

### 2.2 Environmental Monitoring Data

Environmental monitoring sites adjacent to or sharing the same district codes with the subjects’ clinics were used for the sources of environmental data, which came from 47 of the available 66 environmental monitoring sites in Taiwan. Data for the monitored air pollutants (CO, NO_X_, O_3_, PM_10_, and SO_2_) were available for download (in daily and monthly averages) from the Taiwan Air Quality Network (http://taqm.epa.gov.tw/taqm/en/default.aspx). To address possible latency in health effects for the cohort database covering 2007–2011, we retrieved the environmental monitoring data from one year ahead of the period (2006) to 2011.

### 2.3 Data Management and Analysis

Data were divided by age or gender to assess potential effects on AR incidence. The cohort database without personal identification provided the subjects’ clinical locations at the county or district level, which was sufficient for linkage with adjacent environmental monitoring sites.

A logistic regression analysis was conducted to compare the rates between ages or genders, whereas Pearson correlation was performed to determine the relationship between the five air pollutants. We used a dummy variable for each subject’s AR occurrence (1 = first occurrence; 0 = one of two randomly selected pre-AR time points) in a logistic regression analysis to calculate the odds ratios (ORs) for AR occurrence and the levels of air pollution when adjusting variables that may have been effective in incidence (e.g., age or gender). Because air pollution-induced respiratory symptoms were confirmed to be related to exposures for several days to weeks [21,22] and the diagnoses may be registered days after the true occurrences, we used the environmental data from one and two weeks prior to the diagnoses for the pre-incident levels of air pollution in the regression analysis. The levels, which were determined by categorizing the average concentrations with quartiles of the descriptive statistics of the 2007–2011 environmental monitoring data, were Level 1 from the lowest to Q1, Level 2 from Q1 to Q3, and Level 3 from Q3 to the highest. All statistical analyses were performed using SAS 9.2.

## 3. Results

The first AR incidence in Taiwanese preschool children for 2007–2011 was 10.9%, with the boys’ outcome significantly higher than the girls’ (14.2% > 8.28%, OR = 1.83, 95% CI = 1.62–2.09) (Table 1). The rates significantly increased with age from 4.21% to 18.6% as age increased from 2 to 6. This increasing trend should be of concern to parents and caregivers because AR could lead to chronic respiratory diseases or asthma without proper care or treatment.

The statistical results of the daily concentrations of the monitored air pollutants corresponding to the subjects are presented in Table 2. The maximum daily concentrations of the pollutants were below the ambient standards, indicating that air quality during the period in Taiwan was generally acceptable. The mean concentrations, which are considered to be the averages of annual concentrations from 2006 to 2011, were comparable with the annual ambient standards, which were available for PM_10_ and SO_2_. The mean concentration of SO_2_ (4.81 ± 1.85 ppb) was far below its annual standard (30 ppb), showing the efficacy in emission control, whereas that of PM_10_ (56.8 ± 14.4 μg/m^3^) was close to the standard (65 μg/m^3^), suggesting a need for control efforts.

The correlation between the air pollutants is given in Table 3. NO_X_, a byproduct of combustion, was highly correlated with CO and SO_2_ (*r* = 0.851 and 0.809), indicating emission sources of motor vehicles and industry/power plants, respectively, because CO was primarily produced by motor vehicles and SO_2_ was a major combustion product of fuel [23]. NO_X_ was also highly correlated with O_3_ but in an opposite direction (*r* = −0.801), and a similar correlation was found between SO_2_ and O_3_ (*r* = −0.485). The negative association between NO_X_ or SO_2_ and O_3_ suggests the consumption of either precursor of the photochemical smog reaction that generates O_3_. PM_10_ was not significantly associated with any other variables with the exception of SO_2_ (*r* = 0.531), indicating that a certain quantity of PM_10_ in Taiwan was generated from fuel combustion.

Age and gender both affected the AR incidence (as shown in Table 1); thus, we adjusted for those factors when conducting logistic regression analyses. The univariate analysis shows significant ORs for AR occurrence when comparing the higher pre-incident levels of CO or NO_X_ (e.g., Levels 2 and 3) with the low level (Level 1) either one or two weeks prior to the occurrence (Table 4). Regarding other pollutants (O_3_, PM_10_ and SO_2_), the level differences did not significantly affect AR occurrence. We also performed multivariate logistic regression analyses to determine the association between AR and all air pollutants together but excluded CO from the analyses because of its collinearity with NO_X_ (*r* = 0.851). NO_X_ is the only variable that was significantly related to AR with ORs as high as 1.94 (95% CI = 1.41–2.71) for one week before and 1.50 (95% CI = 1.19–2.03) for two weeks before. The pre-incident levels one week before occurrence resulted in higher ORs than that two weeks before, suggesting that AR induced by air pollution should occur within a week. In summary, the relatively high pre-incident levels of traffic-related pollutants (NO_X_ coupled with CO) were likely to lead to AR within the following week.

## 4. Discussion

Our finding that the incidence of first AR was higher for boys than girls is consistent with other previous reports [4,10,11]. As indicated by other studies, more severe bronchial responsiveness and/or airway inflammation for boys than girls could explain this finding [24,25]. Lu *et al*. [11] reported an 8.4% prevalence in Chinese preschool children, which was less than our finding (10.9%); however, the ascending trend with age did not differ from ours. They also noted that age-related accumulative personal exposure to air pollutants was significantly associated with AR, suggesting that prevention from exposure to air pollution helps minimize the likelihood of AR occurrence in children.

Our finding of the association between AR and two air pollutants, CO and NO_X_, in preschool children was consistent with the results from previous Taiwanese studies assessing different ages [4,10], suggesting that AR occurrence in children at all ages in Taiwan should be related to outdoor concentrations of CO and NO_X_. Lee *et al*. [4] noted that outdoor NO_X_ rather than personal NO_X_ was better predictive of AR [26], indicating that NO_X_ itself was likely not the direct cause of AR. Hwang *et al*. [10] noted no biological plausibility for CO to influence the airways, suggesting a role for a CO surrogate. Thus, both previous studies considered that these two pollutants served as surrogates for traffic emissions that may have altered the susceptibility of children to allergens. Several studies focused on the effects of traffic emissions by examining the odds ratios for AR or respiratory symptoms with the residential proximity to major roads. A Korean study found that living less than 75 m from a major road was significantly associated with AR for children [15]; a Polish study indicated that seven-year-old children living within 200 m of a major roadway had a significant OR of 1.4 for nasal symptoms compared with those living within 200–500 m, and an even higher OR compared with those living beyond 500 m [17]. Regarding adults, a Swedish survey showed that living within 100 m of a road with a traffic intensity of >10 cars/min was significantly associated with AR (OR = 1.30) [27]. Based on these results, exposure to more traffic emissions may be more likely to lead to AR or other respiratory symptoms at all ages.

One of the traffic emissions of great concern is diesel exhaust particulate (DEP) [28], which has been found to induce the production of Immunoglobulin E (IgE) antibody and oxidative stress in the airways; that is, DEP may enhance allergic inflammation, sensitize the airways to subsequent allergen exposure, and exacerbate allergic symptoms [18,22]. A recent study indicated a significantly positive association between DEP and aeroallergen sensitization for young children less than four years [29], supporting the role of DEP in respiratory allergies. Additionally, DEP may act as an adjuvant to allergens to enhance the allergic response [30]. A Japanese study demonstrated that no IgE antibody response to an AR-causing allergen in Japan was observed from the allergen immunized mice; however, positive results were shown for those immunized with the allergen together with DEP [31].

Other than the association between AR and traffic-related air pollutants, our results also resembled those of previous Taiwanese studies by demonstrating a negative association between O_3_ and NO_X_ [4,10]. This is not surprising because O_3_ is a secondary product derived from the photochemical smog reaction, which consumes NO_X_ and hydrocarbons in the presence of sunlight to form O_3_ [32]; additionally, Taiwan’s location in the sub-tropic and tropic regions means that it is a favorable place for the photochemical smog reaction to take place to produce O_3_. By integrating these three Taiwanese studies, we conclude that the correlation between air pollutants has been consistent since 1994 and the association between AR and outdoor concentrations of CO and NO_X_ has been identically significant for preschool children, middle school children and adolescents.

In comparing our data with the environmental data of both previous Taiwanese studies [4,10], we found that the annual concentration of O_3_ in Taiwan had gradually increased over the years (reflecting climate change), which resulted in higher temperatures in favor of O_3_ production [32]. Despite no significant relationships between AR and O_3_ in this study, O_3_ is a threat to respiratory health due to its reactivity and irritating effect. However, studies have found a significant association between O_3_ and respiratory allergies [7,9,12], suggesting that the impairing effect of O_3_ should not be overlooked.

There were several limitations to this study. First, we did not have access to personal information and thus could not assess direct AR-triggering effects; using the identification-free cohort database, however, we were able to determine the impacts of outdoor air pollution on AR. Secondly, environmental monitoring data were derived based on the locations of the clinics and not where the patients actually lived because of a lack of access to personal information. Fortunately, this did not appear to present an issue because most Taiwanese who lived in urban areas were believed to go to nearby clinics for minor respiratory diseases, such as colds and AR. Thirdly, PM_2.5_, one of the major pollutants of traffic emissions, was not included in this study because such data were not measured at all environmental monitoring sites. We expect associations between PM_2.5_ and respiratory symptoms to be assessed in the future in Taiwan when PM_2.5_ becomes a regular measure for all environmental monitoring sites.

## 5. Conclusions

The first AR incidence for preschool children in Taiwan was higher in males than females for 2007–2011. CO and NO_X_, which are considered to be traffic-related pollutants, were found to be significantly associated with AR in preschool children during the assessed period in Taiwan. The association is consistent with those derived from previous Taiwanese studies focusing on middle school children, suggesting that traffic emissions in Taiwan need to be more effectively controlled to lower the likelihood of AR occurrence.

## Figures and Tables

**Table 1:** Incidence rates of the first occurrence of allergic rhinitis in preschool children by gender and age for 2007–2011.

Population	Rhinitis Cases (%)	Odds Ratio (95% CI)
Total (*n* = 9960)	1088 (10.9%)	
Gender		
Male (*n* = 4456)	633 (14.2%)	1.84 (1.62, 2.09) *
Female (*n* = 5504)	455 (8.27%)	Referent
Age at first diagnosis (years)		
0–2 (*n* = 3342)	141 (4.21%)	Referent
3 (*n* = 1732)	171 (9.87%)	2.49 (1.97, 3.13) *
4 (*n* = 1803)	210 (11.6%)	2.99 (2.40, 3.74) *
5 (*n* = 1574)	286 (18.2%)	5.04 (4.08, 6.23) *
6 (*n* = 1509)	280 (18.6%)	5.18 (4.19, 6.41) *

* Statistically significant.

**Table 2:** Descriptive statistics of daily concentrations of air pollutants for 2006–2011 in Taiwan.

Air Pollutant	Mean ± SD	Minimum	25th Percentile (Q1)	Median (Q2)	75th Percentile (Q3)	Maximum	Taiwan’s Ambient Standards
CO (ppb)	561 ± 172	150	448	553	618	1,070	9000 ^a^
NO_X_ (ppb)	19.6 ± 5.72	2.33	16.1	20.5	22.9	29.7	50 ^b^
O_3_ (ppb)	27.9 ± 3.85	20.5	25.9	27.7	29.9	41.7	60 ^a^
PM_10_ (µg/m³)	56.8 ± 14.4	21.7	47.9	56.7	65.0	88.1	65 ^b^; 125 ^c^
SO_2_ (ppb)	4.81 ± 1.85	1.90	3.51	4.32	6.11	11.7	30 ^b^; 100 ^c^

CO, carbon monoxide; NO_X_, nitrogen oxides; O_3_, ozone; PM_10_, particulate matter with aerodynamic diameter ≤ 10 μm; SO_2_, sulfur dioxide; SD, standard deviation. ^a^ 8-h average; ^b^ annual average; ^c^ daily average.

**Table 3:** Correlation between daily concentrations of the monitored air pollutants for 2016–2011 in Taiwan.

(*n* = 2191)	CO	NO_X_	O_3_	PM_10_	SO_2_
CO	1.000				
NO_X_	0.851 **	1.000			
O_3_	−0.744 **	−0.801 **	1.000		
PM_10_	0.195	0.320	−0.109	1.000	
SO_2_	0.630 *	0.809 **	−0.485 *	0.531 *	1.000

* *p* < 0.05; ** *p* < 0.01.

**Table 4:** Uni- and multivariate logistic regression analyses between AR occurrence and different pre-incident levels of air pollutants after adjustment for age and gender.

Air Pollutant Level	One Week before AR		Two Weeks before AR	
	**Univariate**	**Multivariate**	**Univariate**	**Multivariate**
(*n* = 3264)	OR (95% CI)	OR (95% CI)	OR (95% CI)	OR (95% CI)
CO				
Level 1	1.00		1.00	
Level 2	1.69 (1.05–1.95) *		1.40 (1.15–1.91) *	
Level 3	1.75 (1.21–2.29) *		1.14 (1.02–1.86) *	
NO_X_				
Level 1	1.00	1.00	1.00	1.00
Level 2	1.67 (1.12–1.94) *	1.83 (1.38–2.53) *	1.33 (1.03–1.75) *	1.50 (1.19–2.03) *
Level 3	1.78 (1.24–2.45) *	1.94 (1.41–2.71) *	1.47 (1.14–1.84) *	1.41 (1.11–1.89) *
O_3_				
Level 1	1.00	1.00	1.00	1.00
Level 2	0.93 (0.64–1.32)	0.95 (0.69–1.16)	1.35 (0.81–1.61)	1.22 (0.81–1.64)
Level 3	0.97 (0.68–1.37)	1.02 (0.70–1.22)	1.27 (0.76–1.70)	0.97 (0.62–1.20)
PM_10_				
Level 1	1.00	1.00	1.00	1.00
Level 2	1.08 (0.75–1.58)	1.14 (0.64–1.32)	0.86 (0.58–1.23)	1.05 (0.72–1.23)
Level 3	1.15 (0.56–1.59)	1.09 (0.42–1.47)	1.12 (0.79–1.45)	1.08 (0.81–1.35)
SO_2_				
Level 1	1.00	1.00	1.00	1.00
Level 2	0.52 (0.36–1.26)	0.74 (0.49–1.16)	1.00 (0.63–1.29)	0.79 (0.61–1.18)
Level 3	0.56 (0.33–1.31)	0.85 (0.68–1.27)	1.05 (0.67–1.33)	0.87 (0.69–1.26)

* *p* < 0.05; OR, odds ratio; ppm, parts per million; ppb, parts per billion.
